# Curbing Ebola infections among healthcare workers in West Africa: unconventional strategies needed

**DOI:** 10.11604/pamj.2014.19.320.5716

**Published:** 2014-11-26

**Authors:** Onyema Eberechukwu Ogbuagu, Arit Ogbuagu

**Affiliations:** 1 Section of Infectious Diseases, Yale University School of Medicine, 135 College Street, Suite 323, New Haven, Connecticut, USA; 2 Yale AIDS Program, Yale University School of Medicine, New Haven, Connecticut, USA

**Keywords:** Ebola infection, healthcare workers, immunity

## To the editors of the Pan African Medical Journal

One of the tragedies of the current ongoing West African Ebola virus outbreak is the large number of healthcare workers (HCWs) who have been infected with the disease. As of October 31, 2014, there were 523 cases of infection among HCWs, and 269 have died [1]. It is well known that infected HCWs may also initiate or propagate nosocomial outbreaks such that disease transmission is amplified. The sad fact is that the unfortunate HCW infections and deaths are largely preventable. However, this tragic misfortune presents an opportunity.

There is evidence that individuals who survive the Ebola virus infection develop protective natural immunity that lasts up to 10 years from the time of infection [2, 3]. In recognition of this, the use of convalescent blood or serum from Ebola survivors, which has been used successfully in the past to treat new infections, was recently endorsed by a World Health Organization (WHO) expert consultation panel to be prioritized as a treatment option in the face of a raging epidemic with limited available therapeutic alternatives [4]. Convalescent blood, a form of passive immunotherapy, has been administered to Ebola patients in the United States as part of a multimodal treatment strategy with comparatively excellent results to date.

It makes sense, therefore, that part of the regional strategy to limit or reduce further infections among HCWs in heavily affected countries, especially those with limited resources, should include encouraging HCW-Ebola survivors who are probably immune to reinfection, and are willing, to “re-engage” in the care of infected patients, especially direct patient care activities. There are now more HCW-Ebola survivors than any other time in history of the disease, and they represent the least vulnerable health care providers. Until an Ebola virus vaccine is available, this proposed strategy, where feasible, should mitigate the risk of disease transmission to non-immune healthcare workers on the front lines of patient care. While one may argue that the use of appropriate personal protective equipment (PPE) should also decrease risk of disease acquisition, as has been observed in “real-life” experiences in Ebola outbreaks, and for varying reasons, PPE use and protection is neither fool-proof nor perfect.

The use of HCWs with immunity to a disease to care for patients suffering from the same is not an entirely novel approach. The strategy has been employed successfully in outbreak situations, to limit the spread of other communicable diseases such as measles [5]. That said, we appreciate and admire every HCW who, in spite of great risk to their personal health, and in uniquely difficult circumstances, have participated in the response to the epidemic. HCWs have made a huge contribution to improved clinical outcomes of patients in this current Ebola outbreak. Control of this epidemic definitely requires thinking “outside the box” and unconventional strategies are sorely needed to stem the tide of rising infections.

## Conclusion

Healthcare workers (HCWs) are at increased risk of acquiring Ebola infection and have been disproportionally impacted by the current West African Ebola outbreak. Survivors of the disease develop protective natural immunity that can prevent reinfection. In resource limited settings, HCW-Ebola survivors can be utilized as valuable front-line providers of care to Ebola patients and thereby reduce risk of disease transmission to non-immune HCWs.
